# The impact of the Vancouver Winter Olympics on population level physical activity and sport participation among Canadian children and adolescents: population based study

**DOI:** 10.1186/s12966-014-0107-y

**Published:** 2014-09-03

**Authors:** Cora L Craig, Adrian E Bauman

**Affiliations:** Public Health, School of Public Health, The University of Sydney, 2006 Sydney, NSW Australia; 201-185 Somerset Street West, K1S2J8 Ottawa, Ontario Canada

**Keywords:** Physical activity, Public health, Evaluation, Epidemiology

## Abstract

**Background:**

There has been much debate about the potential impact of the Olympics. The purpose of this study was to determine if hosting the 2010 Vancouver Olympic Games (OG) encouraged Canadian children to be physically active.

**Methods:**

Children 5–19 years (n = 19862) were assessed as part of the representative Canadian Physical Activity Levels Among Youth surveillance study between August 2007 and July 2011. Parents were asked if the child participated in organized physical activity or sport. In addition, children wore pedometers for 7 days to objectively provide an estimate of overall physical activity. Mean steps/day and percent participating in organized physical activity or sport were calculated by time period within year for Canada and British Columbia. The odds of participation by time period were estimated by logistic regression, controlling for age and sex.

**Results:**

Mean steps were lower during the Olympic period compared with Pre- (607 fewer steps/day 95% CI 263–950 steps/day) and Post-Olympic (1246 fewer steps 95% CI 858–1634 steps) periods for Canada. There was no difference by time period in British Columbia. A similar pattern in mean steps by time period was observed across years, but there were no significant differences in activity *within* each of these periods between years. The likelihood of participating in organized physical activity or sport by time period within or across years did not differ from baseline (August-November 2007).

**Conclusion:**

The 2010 Olympic Games had no measurable impact on objectively measured physical activity or the prevalence of overall sports participation among Canadian children. Much greater cross-Government and long-term efforts are needed to create the conditions for an Olympic legacy effect on physical activity.

## Introduction

There has been much enthusiastic speculation that hosting the Olympic Games (OG) encourages individuals, particularly children, to be more physically active [1,2]. Conducting a mega event such as the Olympics is an opportunity to promote sport and physical activity to the community, and to stimulate community interest, utilise new facilities, and foster sports participation [3]. Typical Olympic “legacies” focus on infrastructure developments, social inclusion, transport systems, and potential economic benefits that might result from hosting an OG [4]. Increased sports club membership among children and adolescents was reported following the Manchester Commonwealth Games in 2002 [3]. A series of studies examined the impact of participation in the Youth OG in Innsbruck in 2012, but only focused on the effects on young athletes and their coaches [5].

Historically, there is little evidence to support the oft-held belief that hosting the Olympic Games promoted increased broader population levels of physical activity among adults and children. In fact, in the reverse has been reported previously, with physical activity levels among adult Australians slightly declining six weeks after the Sydney Olympics in 2000, compared to 1997 and 1999 [6,7]. It is possible that children and youth might be more amenable than adults to a trickle-down effect of OG on participation. The bid for the 2012 London Olympics was based on legacy promises of increasing ‘motivation to be active’ among children [4]. However, no studies have examined the impact of mega events on population levels of physical activity (PA) among young people.

Increasing physical activity among children was a strong focus of the Vancouver 2010 OG. In 2005, the British Columbia (BC) provincial government announced their five major goals for the next decade and among these was “to lead the way in North America in healthy living and physical fitness” [8]. The objective to increase the proportion of British Columbians who were physically active by 20% was linked to the new ActNow BC program as a means to make ‘British Columbia the healthiest jurisdiction to host the Olympic and Paralympic Games’ [9]. BC had previously reported high physical activity participation rates among adults and children, compared to other parts of Canada [10-13] and was among the healthiest provinces in the nation [9]. The launch of ActNow BC prior to hosting the 2010 Winter OG provided children and adults in BC with increased opportunities to become more physically active and to access recreational physical activity and sport through additional infrastructure [9].

In addition to the overall OG media attention, a youth initiative was launched that explicitly linked PA participation with the Olympics. SOGO Active was created by a non-Government organisation, ParticipACTION in 2008 in conjunction with Coca Cola Canada as part of their sponsorship of the 2010 OG. Coca Cola initially partnered with ParticipACTION to foster youth-led groups who then designed and implemented activities to engage other youth in PA [14,15]. These programs helped young Canadians to access facilities, equipment and seed funds provided through a network of ParticipACTION. SOGO Active listed partners in every Canadian Province and Territory, including BC. As an incentive, SOGO Active participants were informed that they were eligible to be one of 1000 Olympic torchbearers based on outstanding peer leadership.

This media promotion of SOGO Active, its link to the 2010 OG, and related Provincial activities provided a unique opportunity to examine whether hosting the Winter Olympic motivated Canadian children and youth to become more physically active from a public health perspective. A Canadian surveillance system collected steps/day as an objective measure of overall PA data nationally, and in the host province of BC; this was used in this study [16] to examine population physical activity levels during and after the 2010 Winter Olympic Games in Vancouver. The central research question is whether hosting the February 2010 Vancouver Winter Olympic Games further increased overall physical activity and the prevalence of sport participation from their initial relatively high levels among children 5–19 years in British Columbia and in Canada more broadly.

## Methods

The Canadian Physical Activity Levels Among Youth study (CANPLAY) is a children’s physical activity surveillance system, it uses objective measures of overall PA (with pedometers) and collects continuous data since 2005 [17]. The methods and data treatment protocols have been described extensively [16,18]. In summary, children aged 5 and 19 years were randomly sampled and recruited into the study, (yielding a representative sample at the national and provincial level). During the recruitment interview, parents were asked if each child participated in organized physical activity or sport (yes/no). The study was explained and parents who agreed to their child’s participation in the pedometer portion of the study (~6,000 families annually) were sent a data collection kit. Participants wore the pedometer for up to 7 consecutive days and were asked to log and report their daily steps. Informed consent/assent was received from all participants and from legal guardians where appropriate. Study protocols were approved by the Human Participants Review Committee of York University for 2007–2011 (#2010-024), and by the Health Canada Ethics Review Board for 2010 and 2011 (#CANPLAY-REB-2010-2012). For this Olympics-focused analysis, all data between August 2007 and July 2011were included. The pedometer was worn for at least 5 days by over 95% of participants. Daily step counts were rounded up or down to 1,000 and 30,000 steps/day if daily values fell outside that range.

Given an approximate one month lag for return of pedometer data, the month of receipt was classified into Pre- (August-November, 2009), Post- (April-July, 2010) and Olympic Games period (December 2009-March 2010, as the Torch Relay and Winter OG spanned the period November 2009 to February 2010. These terms were used to describe the same time periods in earlier years. The mean steps/day across logged days and percent participating in organized physical activity or sport were assessed by time period within year, for Canada and for BC. Differences were computed between the overall mean steps/day during the 2010 Olympic period and the mean steps/day in other time periods within age-sex groups. The prevalence of any participation in organized PA or sport was estimated. The odds of any participation in organized physical activity and sport related to the pre-, post- and OG time periods was estimated by logistic regression, controlling for age and sex. All estimates were computed using SPSS Complex Sample procedures (SPSS version 18, IBM, Chicago Illinois, USA) to weight data and account for the sample design.

## Results

Overall, 19682 boys and girls (5–19 years) participated in the CANPLAY study (Table 1). Of these 1375 lived in BC. Mean steps/day were higher among boys than girls and for younger as compared with older age groups, both overall and separately for boys and girls. This was true for Canada as a whole and for BC. Mean steps/day were generally higher in BC compared with the rest of Canada, regardless of sex and age. However, this difference was not significant for youth aged 15–19 years.

**Table 1:** **Mean steps/day and participation in organized physical activity or sport among participants, August 2007-July 2011**

	**Canada**		**British Columbia**	
	**n**	**Mean steps/day (95% CI)**	**n**	**Mean steps/day (95% CI)**
Pedometer counts				
Total	19682	11241 (11147, 11334)	1375	11880 (11621, 12139)
Boys	9923	11865 (117336, 11994)	680	12501 (12150, 12851)
Girls	9759	10601 (10492, 10712)	695	11265 (10968, 11562)
5-9 years	7386	12038 (11910, 12166)	539	12716 (12397, 13036)
10-14 years	7860	11405 (11277, 11533)	547	11970 (11616, 12324)
15-19 years	4434	9876 (9698, 10056)	289	10361 (9797, 10926)
Boys, 5-10	3712	12651 (12474, 12827)	288	13280 (12859, 13700)
Boys,11-14	4004	12069 (11884, 12255)	251	12696 (12151, 13241)
Boys, 15-19	2205	10432 (10177, 10687)	141	10729 (9951, 11506)
Girls, 5-10	3674	11415 (11250, 11579)	251	12039 (11615, 12464)
Girls,11-14	3212	10908 (10742, 11074)	238	11502 (11062, 11982)
Girls, 15-19	2873	9464 (9277, 9650)	206	10253 (9726, 10780)
Participation in organized physical activity or sport				
Total	23314	75.7 (74.8, 76.6)	1655	77.2 (74.5, 79.7)
Boys	11799	76.9 (75.8, 78.1)	812	79.1 (75.5, 82.2)
Girls	11515	74.4 (73.1, 75.7)	843	75.4 (71.9, 78.6)
5-9 years	8279	84.6 (83.3, 85.7)	602	86.0 (82.5, 88.9)
10-14 years	9090	80.7 (79.5, 81.9)	642	82.2 (78.7, 85.3)
15-19 years	5943	58.9 (57.0, 60.7)	411	59.4 (53.9, 64.6)
Boys, 5–9 years	4204	84.4 (83.0, 86.1)	323	85.3 (80.2, 89.2)
Boys,10-14 years	4638	81.5 (79.9, 83.1)	299	84.7 (79.7, 88.6)
Boys, 15–19 years	2955	61.8 (59.4, 64.3)	190	62.1 (54.2, 69.4)
Girls, 5–9 years	4075	84.5 (82.8, 86.1)	279	86.9 (81.9, 90.6)
Girls,10-14 years	3621	82.1 (80.3, 83.8)	273	82.2 (77.0, 86.4)
Girls, 15–19 years	3819	59.5 (57.2, 61.8)	291	61.4 (55.1, 67.4)

The prevalence of reported participation in *organized* physical activity or sport was also higher among boys than girls and younger compared with older age groups. A decrease in sport participation rate by age was evident among both boys and girls (Table 1, lower panel). Although the prevalence was slightly higher in BC than nationally, this difference was not significant. Further, differences in organized PA and sport participation rates by age and sex in BC were similar to the national pattern.

### Participation in physical activity (mean steps/day)

**Figure 1:**
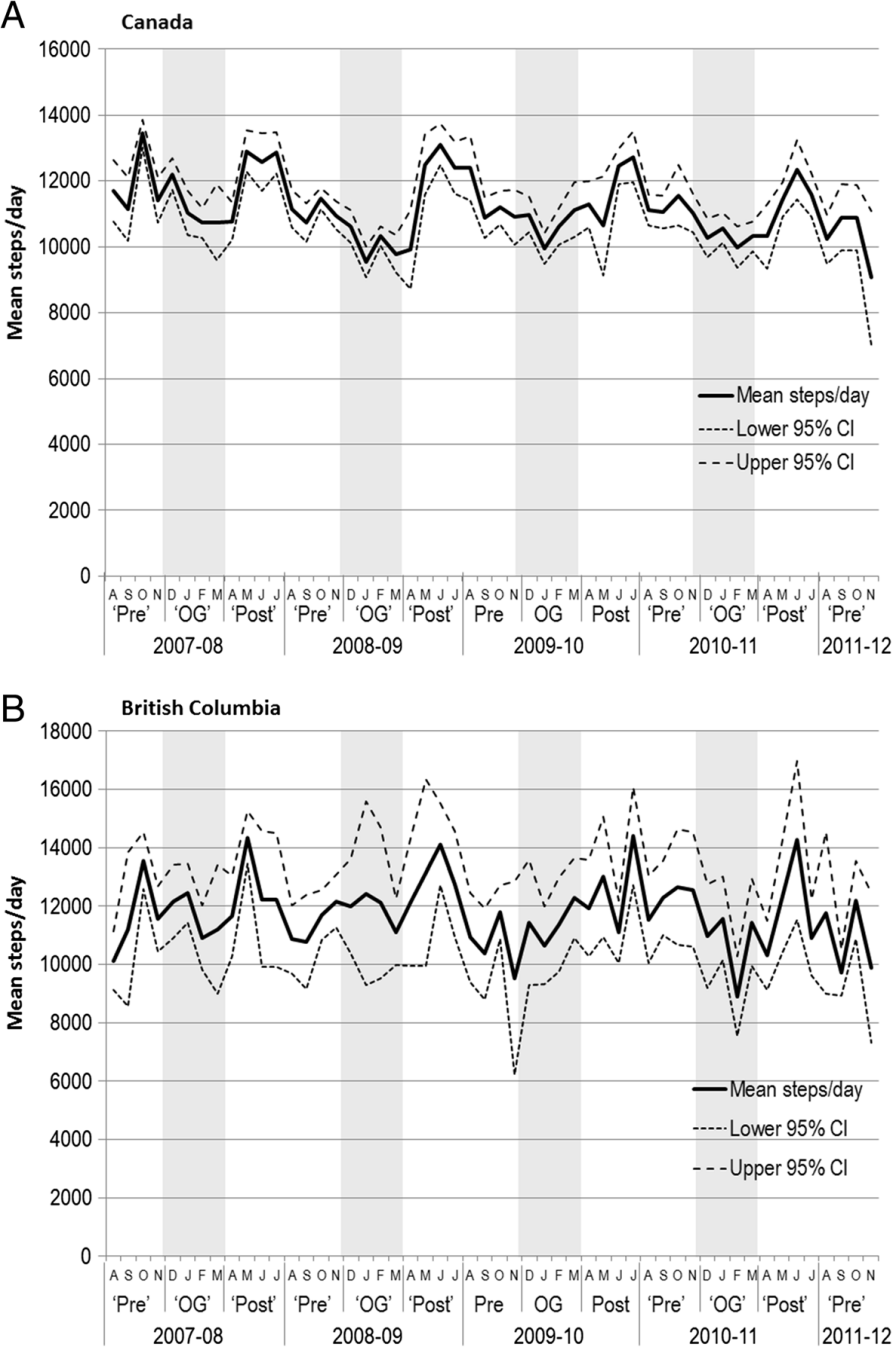
**Mean steps/day by month, Canada and British Columbia.** ‘Pre’ A S O N: August to November. ‘OG’ D J F M: December to March. ‘Post’ A M J J: April to July.

There was considerable seasonal variation in mean daily steps by month, with step counts higher in late-spring to summer (May-July) than during the winter months (December-March). This pattern was less pronounced in British Columbia (Figure 1B) than in Canada overall (Figure 1A).

**Figure 2:**
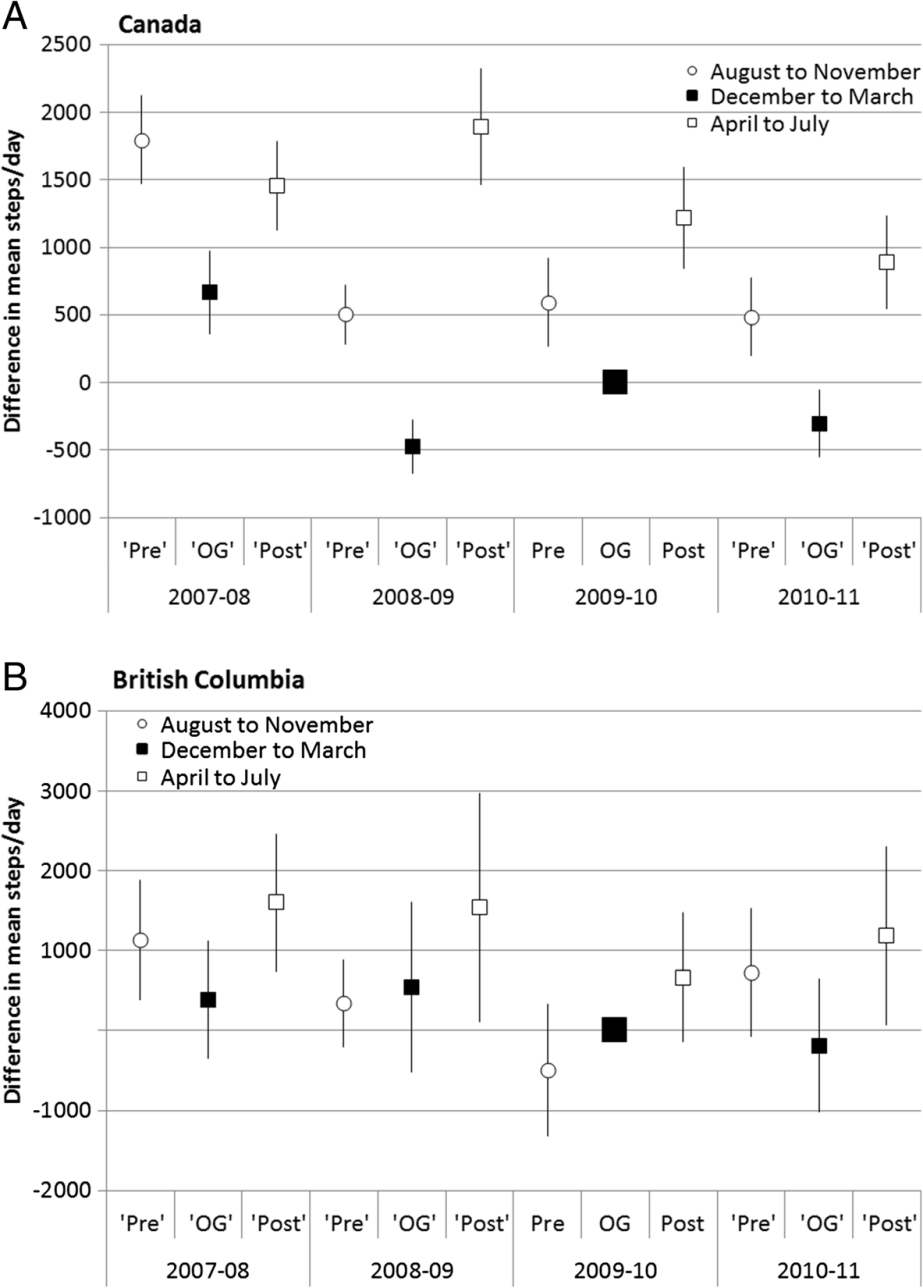
Difference in steps/day compared to mean steps/day* during the 2010 Olympic period (Torch relay, Olympics and Paralympics), Canada and British Columbia. *controlling for age and sex.

Figure 2 (Panel A) shows the difference in steps/day nationally compared with data during the Olympic period (controlling for age and sex). Each year, mean steps were lower during the Olympic period compared with Pre- and Post-Olympic periods with one exception. In 2009, daily steps in the Pre-Olympic period (August to November) did not differ from steps during the Olympic period (December 2009-March 2010). PA during the Olympic period did not differ from estimates derived from the same months in other years; PA during the Post-Olympic period (April to July 2010) did not differ from estimates in the Spring-Summer period in other years. Overall, PA differed among the Pre-, Post- and Olympic periods across years, but there were no secular differences in PA *within* each of these periods between years except for higher PA during the Pre-Olympic period in 2007/08 compared with other years. We also noted lower PA during the 2011 Post-Olympic period compared with the same period in 2009. Results were similar for boys and girls and by age group Canada-wide [data not shown].

The general pattern for PA by period in BC was similar to all of Canada data (Figure 2B), with one significant difference, in that boys reported higher PA during the 2008 Pre-Olympic period compared with other years [data not shown].

### Participation in organized physical activity or sport

**Figure 3:**
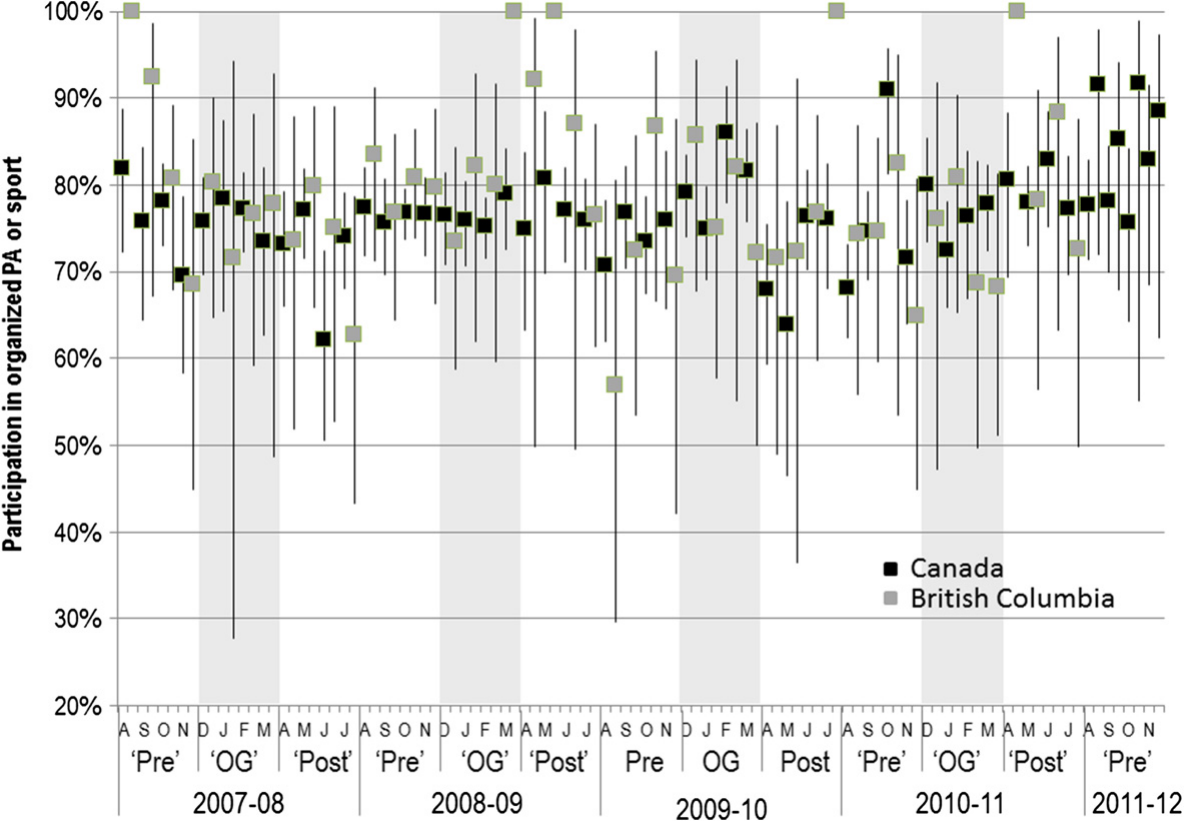
**Participation in organized physical activity or sport by month.**

**Figure 4:**
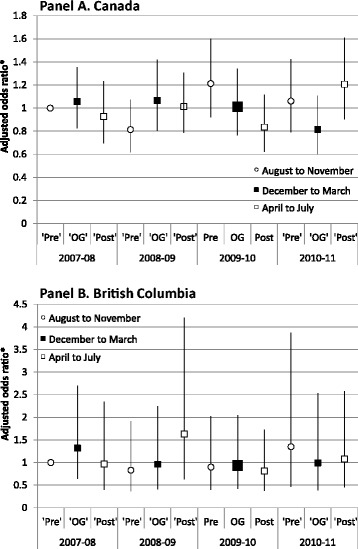
**Relative likelihood of participating in organized physical activity or sport over time, compared to that in August-October 2007, Canada and British Columbia.** *adjusted for age and sex.

The prevalence of participation in organized physical activity or sport is shown in Figure 3. Rates of reported participation varied from 62% in June 2008 to a high of 91% in October 2010 across Canada. Participation rates were similar for British Columbia and ranged from 63% in June 2008 to 92% in October 2010. Participation rates in organized physical activity or sport did not differ during the Olympic time period across the four years of the study in BC or nationwide. In general, there was no difference between participation rates in the Pre- and Post-Olympic periods compared with the Olympic periods. The exception was during the Olympic year (2010), where the participation rate in organized physical activity or sport at the national level was lower during the Post-Olympic months (72%, 95% CI 68-76%) than during the Olympic months (79%, 95% CI 77-82%). By and large, these results were similar for boys and girls and for all age groups. The exception was among boys where the national rate was lower during the 2010 Post-Olympic period (April-July 2010: 69%) compared with the Olympic period (December 2009-March: 81%). Adjusting for age, sex and parental education, there was no difference in the likelihood of participating in any organized physical activity or sport by time period within or across years compared with the Pre-Olympic period for 2008 (Figure 4, Panels A and B).

## Discussion

This study provides evidence to corroborate previous reports that simply hosting an Olympic Games does not enhance population PA levels in those who reside in the host nation or in the vicinity of the Games by extending this finding to young people [2,6,7]. In this study, we note that objectively measured PA levels (steps/day) of Canadian children and youth, did not differ before, during or after the 2010 Winter Olympic Games at the national level or in BC, other than variation attributed to seasonal patterns. Furthermore, there was no difference in participation rates in organized PA or sport across all study periods.

There have been numerous reports that have anticipated increases in population-level sport and physical activity participation following the Olympic Games [1,2]. However, systematic reviews have not found any substantive evidence of an Olympic ‘trickle down’ effect to motivate the population to become more active [19,20]. Some evidence of sports facilities usage was reported after the Manchester Commonwealth Games [3], but no change in overall physical activity was assessed; similarly, positive comments, in the absence of any population surveillance data, were made following the Athens and Beijing Olympics [21,22].

A recent study examined youth sports participation in the years leading up to the London Olympics, and identified a variable pattern, showing no clear increase or decrease between 2006 and 2009 in England [23]. This British paper described the ‘myths of legacy’ for Olympic Games as often promoted by ‘politicians keen to grandstand’ (to hide the UK crisis in youth sports and physical education) [23]. Given this, it is unlikely that a legacy effect for youth as a result of the London Olympics would occur without considerable concerted and sustained investment. Others have examined adult sport participation [24] and reported an increase in ‘any sport participation’ in England between 2005-2012; although this may reflect an increase in the sport sector, it may not be sufficient to enhance health, if the increase in sport is not regular or widespread enough to increase the proportion of populations meeting minimal physical activity guidelines.

National data from the Canadian Community Health Survey (CCHS) on self-reported participation in leisure-time PA among teenagers corroborate our findings [12]. While we acknowledge the limitations of self-report PA data in this age group, the CCHS showed no change in overall PA among boys over the time frame of our study. In girls, the CCHS reported a slight (but non-significant) increase from 64% in 2007/2008 to 67% in 2011/12 in the proportion of girls who reported engaging in moderate-intensity PA. When confined to BC, the CCHS data demonstrated a non significant increase followed by non significant decrease in PA for both teenage boys and girls over a similar time frame. Data confined to the Vancouver Health Region showed no significant changes over time among adolescents (CCHS 105–0502). Thus, CCHS data corroborated the finding that the 2010 Olympic Games had no substantial measureable impact on overall PA nationally, in BC or locally in the Vancouver area.

There were a number of Canadian programs targeting children and youth prior to the 2010 Olympics, including Provincial PA strategies, with a major emphasis on increasing children’s PA [25]. A national Children’s Fitness Tax Credit had been legislated in 2007 to help defray the cost of children’s enrolment in organized PA and sport. Also, in 2007, ParticipACTION aired a campaign to raise awareness among parents about children’s low PA levels [26]. ParticipACTION partnered with Coca-Cola Canada to launch the SOGO Active provided programs and incentives to promote PA among Canadian youth [14,15]. After the OG, a legacy phase continued to promote youth leadership and to offer seed grants to increase access to PA opportunities.

Five years of policy initiatives in BC preceded the Vancouver OG. In 2004, The BC Healthy Living Alliance—a coalition of 4300 health and recreation professionals—developed “The Winning Legacy” to address chronic disease risk factors [9]. The plan was adopted by the BC Government as Healthy BC 2010 and later renamed ActNow BC. The Alliance also developed a physical activity strategy in 2007 (BCHLA 2007) and a framework for designating “Active Communities” [8].

The media coverage of the Games itself was unprecedented; television coverage was double that of the 2006 Olympics and triple that of the 2002 Games, and reached 99% of Canadians, and allowing high community exposure [27]. The Olympics resulted in additional built infrastructure to support recreational physical activity and sport in the Vancouver region and provided Provincial level funding for Action Schools! BC program in elementary schools [27].

Thus, the lack of effects on activity among children was surprising, given the BC history of physical activity policy and program development leading up to the Vancouver Winter Olympics. The lack of impact may have been due to a ceiling effect with high rates of physical activity, limiting the possibility of further improvements; but national data including less active Provinces also showed no Olympic effect. This evaluation only focused on PA and sport participation rates, and there was no triangulation through qualitative and other research methods to assess intermediate outcomes. For example Action Schools! BC, an initiative of ActNow BC, increased objectively measured physical activity (among boys) and fitness levels in intervention schools relative to comparison schools prior to the OG [28]. Therefore, the confounding effects of antecedent and concurrent programs such as Act Now BC and ParticipACTION may have attenuated any observed Olympic effects.

Interestingly, these optimal ‘conditions’ included longer term policy consistency in BC than usually occurs in the years leading up to Olympic Games. The lack of a measureable OG effect after the 2010 Games may indicate that a longer time period is required for the development and implementation of policy and programs to influence overall population levels of PA. Elsewhere, no sustained physical activity programs occurred prior to Sydney, Beijing or Athens Olympics [4], and policy inconsistency and variations to evaluation methods characterised the peri-Olympic period in the London 2010 OG [29]. It seems the enthusiasm for legacy findings on physical activity remains unproved [20], and the expected impact on sports participation (for example following the Glasgow 2014 Commonwealth Games) [30], may be unduly optimistic. Further, the ‘euphoria’ from the ‘festival effect’ surrounding the Olympic Games may induce community feelings of wellbeing, but will not translate into population participation without much stronger community supports [31]. It is time to reject the idea that hosting Olympic Games can spark population level increases in PA. The OG may serve as a platform to promote the idea of increasing PA, but much more concerted public health intervention is required to achieve increased PA at the population level. Future planning and research efforts to assess the impact of OG and other large scale mass events should be guided by a logic model, such as that proposed by Bauman and Murphy [4], to guide strategy development, assess changes in various components of the strategy and measure the overall impact on population PA levels.

This study had several methodological strengths as population-level program evaluation. These included using the unique CANPLAY surveillance system that employs objectively measured PA (steps/day) [16]. Pedometer data measures total PA and best represents ambulatory physical activity. Pedometers are limited in accurately measuring water-based (e.g., swimming) and sliding activities (e.g., ice hockey, Alpine skiing). These measurement issues, and survey response rates are likely to have been non-differential over time. Furthermore, the lack of changes in pedometer-determined PA was corroborated by external self-report data that permitted the inclusion of non ambulatory PA. The study was not an *a priori* evaluation, and was uncontrolled, but did assess representative population samples across multiple time points, starting well before the Vancouver games. The data reported here were of sufficient sample size, and used both objective pedometer data and parental-report data, and the findings were corroborated by self-reported PA participation from the CCHS, an unrelated Canadian health surveillance system.

## Conclusion

In summary, the 2010 Olympic Games had no apparent impact on the objectively measured steps/day (which is a known indicator of total PA) or on increasing the prevalence of reported sport participation of Canadian children. Despite the enthusiasm linking mass sporting events and physical activity [27] there is a clear need for much greater policy commitment and consistency, better synergies between public health and community efforts, mass communication campaigns to change social norms about being active, and building on the Olympics-related infrastructure development and community interest. A much stronger investment and partnership across sectors and agencies is required, with careful evaluation of impact at the population level [3]. Despite having local programs and supportive policy in place through ActNow BC, there was no demonstrable effect on children’s physical activity and sport in BC following the Vancouver Olympics. A much longer sustained and synergistic approach before and after any Olympic Games seems an essential, and as yet unrealised precursor to observing population-level legacy effects on increasing children’s physical activity and sport participation.

## Abbreviations

BC: British Columbia
CANPLAY: Canadian Physical Activity Levels Among Youth study
CCHS: Canadian Community health Survey
OG: Olympic Games
PA: Physical activity
